# Sexual dysfunction associated with antidepressants and antipsychotics: a structured narrative review of mechanisms, assessment, and personalized management

**DOI:** 10.3389/fpsyt.2026.1865752

**Published:** 2026-07-21

**Authors:** Djendji Siladji, Nemanja Maletin, Nikola Denda, Milica Knežević, Milica Paut Kusturica, Dušan Kuljančić, Aleksandar Rašković

**Affiliations:** 1 Clinic for Psychiatry, University Clinical Center of Vojvodina, Novi Sad, Serbia; 2 Faculty of Medicine, University of Novi Sad, Novi Sad, Serbia; 3 Clinic of Internal Oncology, Oncology Institute of Vojvodina, Sremska Kamenica, Serbia; 4 Clinic for Eye Diseases, University Clinical Center of Vojvodina, Novi Sad, Serbia; 5 Clinic for Gastroenterology and Hepatology, University Clinical Center of Vojvodina, Novi Sad, Serbia

**Keywords:** adherence, antidepressants, antipsychotics, psychopharmacology, sexual dysfunction

## Abstract

Sexual dysfunction is a common and clinically significant problem in patients treated with antidepressants and antipsychotics, yet it remains under-recognized in routine psychiatric practice. Its relevance extends beyond tolerability, as it may adversely affect quality of life, intimate relationships, self-esteem, and long-term adherence. A major clinical and methodological challenge is that sexual dysfunction frequently precedes pharmacotherapy, particularly in depressive and psychotic disorders, making it essential to distinguish illness-related impairment from treatment-emergent dysfunction. This narrative review provides an integrated overview of sexual dysfunction associated with antidepressants and antipsychotics, with emphasis on mechanisms, clinical assessment, and management. We summarize current evidence on the comparative risk across major drug classes and selected individual agents, highlighting the generally higher burden associated with strongly serotonergic antidepressants and prolactin-elevating antipsychotics, as well as the relatively more favorable profiles of selected newer or prolactin-sparing compounds. We further examine the principal serotonergic, dopaminergic, endocrine, autonomic, and vascular mechanisms involved, and discuss their relevance to the different domains of sexual functioning. Finally, we review practical strategies for routine care, including baseline evaluation, direct questioning, validated rating instruments, prevention through drug selection, dose adjustment, switching, and adjunctive interventions. Sexual dysfunction should be regarded as a meaningful clinical outcome in psychopharmacology, and its systematic assessment should be incorporated into routine treatment planning and follow-up.

## Introduction

Sexual dysfunction is common in patients with depressive and psychotic disorders and may be present before treatment, emerge during pharmacotherapy, or persist despite improvement of the underlying psychiatric condition (1–4). Its causes are usually multifactorial and may include illness-related symptoms, psychotropic medication, comorbid medical conditions, substance use, relational factors, endocrine disturbances, and their interaction (1–6).

Its clinical importance extends beyond tolerability. Impairment of desire, arousal, orgasm, ejaculation, genital response, or sexual satisfaction may reduce quality of life, affect intimate relationships and self-esteem, and contribute to poor adherence or treatment discontinuation (1, 3–6). Because patients often do not report sexual adverse effects spontaneously, direct questioning and validated instruments are needed for reliable detection (5–8).

Antidepressants and antipsychotics differ substantially in their sexual tolerability profiles. Strongly serotonergic antidepressants, particularly SSRIs and venlafaxine, are consistently associated with a higher risk of treatment-emergent sexual dysfunction, whereas agents with non-serotonergic or multimodal mechanisms, including bupropion, mirtazapine, vortioxetine, agomelatine, moclobemide, and related lower-risk options, generally appear more favorable, although the strength of evidence varies across drugs (1, 2, 5, 7, 9, 10). Among antipsychotics, sexual dysfunction is linked to dopamine D2 blockade, hyperprolactinemia, reduced gonadal hormone activity, autonomic effects, sedation, metabolic burden, and illness-related psychosocial factors (3, 4, 6, 11, 12). Prolactin-elevating agents such as risperidone, paliperidone, amisulpride, and many first-generation antipsychotics generally have a less favorable profile, while prolactin-sparing or partial dopamine agonist agents may be preferable when sexual tolerability is a major clinical priority (3, 4, 6, 11, 12).

This structured narrative review summarizes current evidence on sexual dysfunction associated with antidepressants and antipsychotics, with emphasis on baseline versus treatment-emergent dysfunction, comparative drug-related risk, mechanisms, clinical assessment, and personalized management strategies.

## Methods

This article was designed as a structured narrative review of sexual dysfunction associated with antidepressant and antipsychotic treatment. Its purpose was to provide a clinically oriented synthesis of mechanisms, assessment, comparative sexual tolerability, and management strategies. It was not intended as a systematic review or meta-analysis and does not claim systematic comprehensiveness.

PubMed/MEDLINE, Scopus, Web of Science, and Google Scholar were searched for relevant literature published up to May 2026. Search terms included combinations of sexual dysfunction, treatment-emergent sexual dysfunction, antidepressant-induced sexual dysfunction, antipsychotic-induced sexual dysfunction, psychotropic-related sexual dysfunction, SSRI, SNRI, post-SSRI sexual dysfunction, hyperprolactinemia, prolactin-sparing antipsychotics, bupropion, vortioxetine, vilazodone, agomelatine, moclobemide, tianeptine, aripiprazole, brexpiprazole, cariprazine, lurasidone, and sexual function assessment.

Priority was given to systematic reviews, meta-analyses, randomized or controlled studies, large observational studies, pharmacovigilance analyses, and clinically relevant reviews. Sources were selected according to their relevance to psychotropic-related sexual dysfunction, baseline dysfunction in depressive or psychotic disorders, biological mechanisms, assessment instruments, or management strategies.

**Figure 1:**
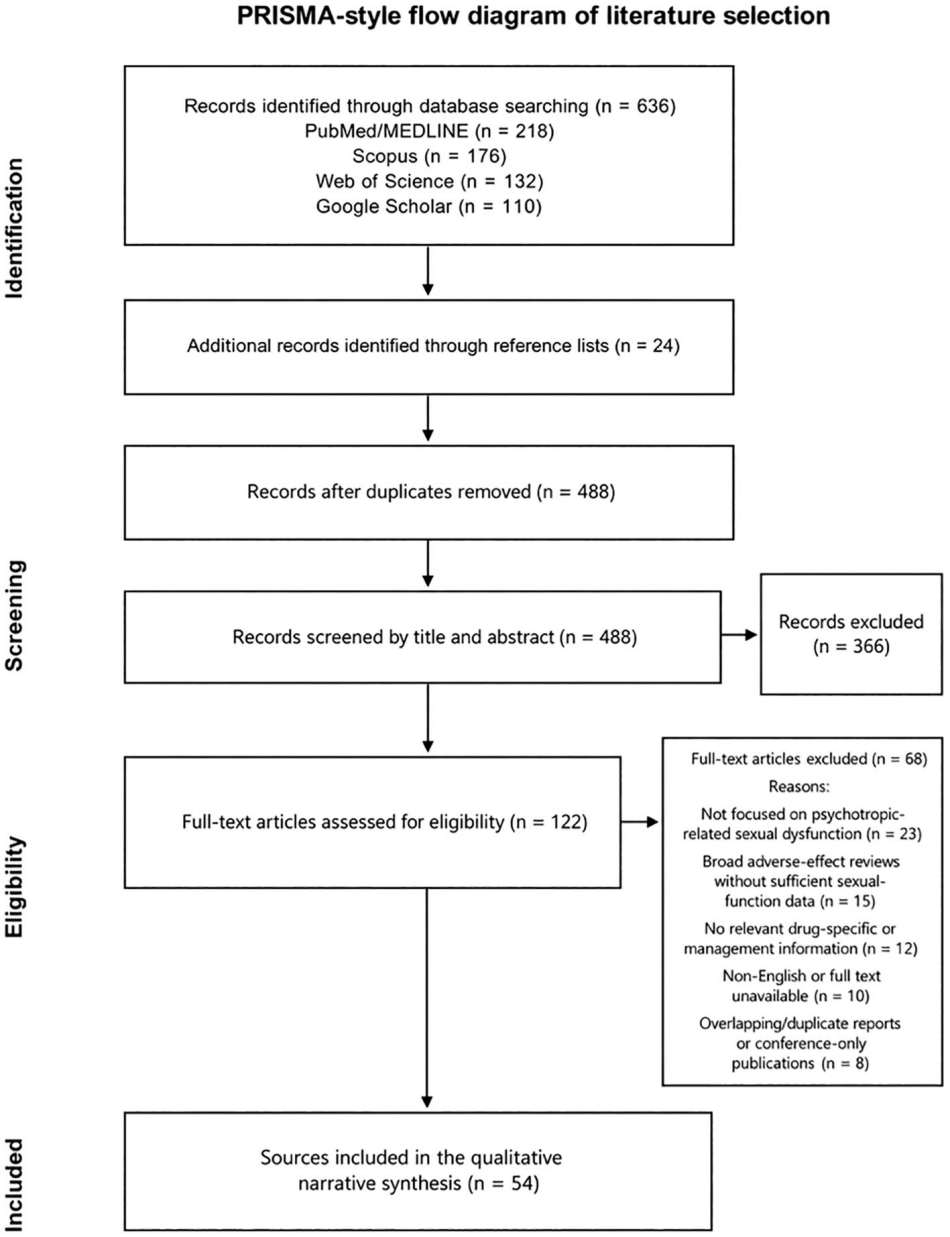
Flow diagram of source identification and selection for this structured narrative review.

No formal risk-of-bias assessment or quantitative synthesis was performed. **Figure 1** is included only to transparently illustrate source identification and selection for this structured narrative review; it does not indicate full PRISMA compliance. The diagram reports 54 evidence sources included in the narrative synthesis, while two additional references relate to reporting methodology and were not counted as included evidence sources (13, 14).

### Sexual dysfunction before treatment: disentangling illness-related and treatment-emergent symptoms

Sexual dysfunction is often present before pharmacotherapy is initiated. In major depressive disorder, impaired desire, arousal, orgasm, and satisfaction may reflect core depressive symptoms, while sexual dysfunction may also contribute to depressive burden and reduced quality of life (1, 2, 7–9).

A similar attribution problem exists in psychotic disorders, where negative symptoms, depressive symptoms, social withdrawal, impaired intimacy, stigma, and illness severity may compromise sexual functioning independently of antipsychotic exposure (3, 4, 10, 11).

Therefore, sexual symptoms emerging during treatment may represent persistence of pre-existing dysfunction, partial improvement with residual impairment, true treatment-emergent worsening, or a combination of these processes. Comparative tolerability data are most interpretable when baseline sexual function, illness severity, and functional status are considered rather than assuming medication causality automatically (1–4).

### Antidepressants and sexual dysfunction: comparative risk across drug classes and individual agents

Antidepressant-associated sexual dysfunction is best understood as a class-related but not class-uniform adverse effect. The following comparisons should be interpreted as a pragmatic clinical synthesis rather than a formal quantitative ranking, because the strength of direct sexual-function evidence differs substantially across drug classes and individual agents. The highest risk is consistently linked to strong serotonergic activity, particularly SSRIs, venlafaxine, and clomipramine, whereas agents with predominantly non-serotonergic, noradrenergic-dopaminergic, melatonergic, reversible MAO-A inhibitory, or multimodal mechanisms generally show a more favorable profile (1, 2, 5, 12, 15). Direct comparisons should nevertheless be interpreted cautiously because studies differ in baseline assessment, definitions of sexual dysfunction, duration, population, dose, and use of validated instruments (1, 2, 5).

SSRIs remain the most clinically important antidepressant group in this context. Paroxetine is often identified as a particularly unfavorable agent, while citalopram, escitalopram, fluoxetine, and sertraline also carry substantial risk for reduced desire, arousal difficulties, delayed orgasm, and anorgasmia (1, 2, 12, 15, 16). Venlafaxine frequently resembles SSRIs in sexual tolerability, especially at lower to moderate doses where serotonergic effects predominate. Duloxetine and desvenlafaxine appear more variable and should be described as intermediate rather than clearly low-risk agents (1, 2, 17, 18).

Tricyclic antidepressants are heterogeneous. Clomipramine is the main high-risk agent, especially for orgasmic delay or anorgasmia, whereas other tricyclics appear intermediate and are influenced by anticholinergic and adrenergic effects (1, 2, 5, 12). By contrast, bupropion has the most consistent evidence for a low rate of treatment-emergent sexual dysfunction, plausibly because of its noradrenergic-dopaminergic mechanism (1, 2, 12, 16). Mirtazapine is generally less sexually disruptive than SSRIs and venlafaxine, although sedation, weight gain, and metabolic effects may limit its overall tolerability (1, 2, 5).

Among newer or less frequently discussed antidepressants, vortioxetine has comparatively favorable evidence, including improvement in sexual functioning after switching from an SSRI in patients with SSRI-related dysfunction (19). Vilazodone may also have a lower sexual burden than conventional SSRIs, but the evidence base is smaller (20). Agomelatine, moclobemide, and tianeptine also belong to the group of antidepressants with comparatively favorable sexual tolerability, although the strength of evidence differs across agents. Agomelatine and moclobemide have been associated with low rates of sexual dysfunction in comparative and review-level evidence, while tianeptine is usually considered potentially favorable but less robustly characterized (21–26).

Overall, the most clinically useful hierarchy is pragmatic: SSRIs, venlafaxine, and clomipramine are higher-risk options; duloxetine, desvenlafaxine, and most non-clomipramine tricyclics are intermediate; and bupropion, agomelatine, moclobemide, mirtazapine, vortioxetine, vilazodone, and possibly tianeptine are relatively more favorable, with the strength of evidence differing across agents (1, 2, 5, 12, 19–26). This hierarchy should guide prescribing only after considering indication, prior response, comorbidity, patient preference, and the clinical importance of preserving sexual functioning.

### Antipsychotics and sexual dysfunction: prolactin, receptor pharmacology, and comparative risk

Antipsychotic-associated sexual dysfunction is common and clinically relevant because it affects quality of life, intimate functioning, fertility-related concerns, and adherence (3, 4, 6, 27, 28). Comparative statements in this section reflect the overall pattern of available evidence rather than a formal ranking, as direct sexual-function data are strongest for some agents and more limited, indirect, or mechanistically inferred for others. As with antidepressants, baseline illness-related dysfunction must be distinguished from treatment-emergent dysfunction. Negative symptoms, depressive symptoms, social withdrawal, stigma, impaired intimacy, and illness severity may all reduce sexual functioning independently of antipsychotic exposure (3, 4, 10, 11).

The most important mechanism is D2 blockade in the tuberoinfundibular pathway, leading to hyperprolactinemia and secondary hypogonadism. This mechanism is especially relevant for risperidone, paliperidone, amisulpride, and many first-generation antipsychotics, which are therefore among the least favorable options when sexual tolerability is a priority (3, 4, 6, 27, 28). However, prolactin is not the only pathway. Dopaminergic blockade may reduce reward and desire; alpha-1 adrenergic effects may impair erection or ejaculation; anticholinergic effects may impair arousal and lubrication; and sedation, weight gain, and metabolic burden may indirectly reduce sexual activity and satisfaction (3, 4, 6).

Aripiprazole has the strongest direct clinical evidence for a comparatively favorable prolactin and sexual-function profile, both as monotherapy and as an adjunctive strategy for antipsychotic-induced hyperprolactinemia (6, 28). Brexpiprazole and cariprazine share partial dopamine D2 agonist activity and are generally prolactin-sparing; however, their sexual tolerability is supported by substantially less direct evidence and should therefore be interpreted more cautiously (29–32). Lurasidone is pharmacologically distinct from these partial agonists but has a relatively favorable prolactin and metabolic profile. Available sexual-function data are limited and less mature than those for aripiprazole (33, 34).

A practical hierarchy is therefore: risperidone, paliperidone, amisulpride, and many first-generation antipsychotics as higher-risk agents; olanzapine and clozapine as intermediate or context-dependent agents; and aripiprazole, quetiapine, ziprasidone, brexpiprazole, cariprazine, and lurasidone as relatively more favorable options, acknowledging that evidence strength differs substantially across compounds (3, 4, 6, 27–35). Key comparative features are summarized in **Table 1**.

**Table 1:** Comparative sexual burden of antidepressants and antipsychotics: mechanisms, evidence base, and clinical implications.

Drug/class	Main mechanism relevant to sexual dysfunction	Comparative sexual burden*	Evidence base	Main clinical implication
SSRIs	Increased serotonergic tone; 5-HT2A/5-HT2C-mediated inhibition	Higher	Strong comparative clinical evidence	Orgasmic delay and anorgasmia are common
Venlafaxine and clomipramine	Predominantly serotonergic activity	Higher	Strong to moderate comparative evidence	Often resemble SSRIs in sexual burden
Duloxetine and desvenlafaxine	Combined serotonergic and noradrenergic effects	Intermediate	Moderate comparative evidence	Not consistently low-burden options
Other tricyclic antidepressants	Mixed serotonergic, adrenergic, and anticholinergic effects	Intermediate	Moderate evidence	Effects vary across individual agents
Bupropion	Noradrenergic-dopaminergic activity without marked serotonin reuptake inhibition	Relatively lower	Moderate to strong comparative evidence	Often preferred when sexual tolerability is prioritized
Mirtazapine	5-HT2/5-HT3 antagonism and noradrenergic activity	Relatively lower	Moderate comparative evidence	Sedation and weight gain may still affect sexual functioning
Vortioxetine	Multimodal serotonergic activity	Relatively lower	Moderate direct clinical evidence	Useful option after SSRI-related dysfunction
Vilazodone	SSRI plus partial 5-HT1A agonism	Potentially lower	Limited to moderate direct evidence	Evidence remains less extensive than for SSRIs
Agomelatine and moclobemide	Melatonergic/5-HT2C antagonism or reversible MAO-A inhibition	Potentially lower	Limited to moderate evidence	Generally favorable profile, but comparative data vary
Tianeptine	Non-SSRI mechanism	Potentially lower	Limited direct evidence	Sexual-function evidence remains sparse
First-generation antipsychotics	Strong D2 blockade, prolactin elevation, autonomic effects	Higher	Strong clinical and endocrine evidence	Desire, erection, and orgasm may be affected
Risperidone, paliperidone, and amisulpride	D2 blockade with substantial prolactin elevation	Higher	Strong clinical and endocrine evidence	Hyperprolactinemia-related dysfunction is prominent
Olanzapine and clozapine	Mixed receptor effects; sedation and metabolic burden	Intermediate/context-dependent	Moderate evidence	Lower prolactin burden does not ensure sexual tolerability
Quetiapine and ziprasidone	Lower prolactin liability; sedative or autonomic effects	Relatively lower	Moderate evidence	Usually preferable to prolactin-raising agents
Lurasidone	Relatively favorable prolactin and metabolic profile	Potentially lower	Limited direct evidence	Direct sexual-function evidence remains limited
Aripiprazole	Partial D2 agonism; prolactin-sparing effects	Relatively lower	Strong direct clinical evidence	Best-supported lower-burden antipsychotic option
Brexpiprazole and cariprazine	Partial D2 agonism; generally prolactin-sparing	Potentially lower	Limited direct evidence	Pharmacologically plausible, but less studied than aripiprazole

*Comparative sexual burden reflects an overall clinical synthesis and should not be interpreted as a fixed quantitative risk estimate. Evidence strength differs across agents: some conclusions are supported by comparative clinical studies, whereas others rely mainly on smaller studies, *post-hoc* analyses, pharmacovigilance findings, endocrine profiles, or mechanistic inference. Drug selection should also consider psychiatric indication, efficacy, dose, prior response, comorbidity, concomitant medication, and patient priorities.

### Mechanisms of psychotropic-related sexual dysfunction: serotonergic, dopaminergic, endocrine, autonomic, and vascular pathways

Psychotropic-related sexual dysfunction is mechanistically heterogeneous and cannot be reduced to a single receptor or endocrine abnormality. It reflects the convergence of central neurotransmitter effects, neuroendocrine changes, autonomic interference, peripheral vascular mechanisms, and broader illness- or treatment-related effects on motivation, energy, and reward processing (3, 6). A clinically useful framework should therefore remain linked to the main domains of sexual function: desire, arousal, orgasm or ejaculation, genital sensation, and overall sexual satisfaction.

The serotonergic pathway is central to antidepressant-associated sexual dysfunction. Increased synaptic serotonin, particularly through post-synaptic 5-HT2A and 5-HT2C receptor stimulation, may suppress sexual desire, impair arousal, and delay or inhibit orgasm and ejaculation (4, 5, 36). These effects are mediated partly through reduced dopaminergic transmission in reward-related circuits and partly through peripheral or spinal inhibition of ejaculatory reflexes, which helps explain the characteristic orgasmic delay associated with SSRIs and other strongly serotonergic antidepressants (4, 12, 15, 37). Conversely, agents that avoid strong serotonin reuptake inhibition or combine it with mechanisms such as 5-HT2 antagonism, dopaminergic/noradrenergic activity, or multimodal serotonergic effects tend to show more favorable sexual tolerability (1, 2, 5, 19, 20).

Dopaminergic mechanisms are especially relevant for sexual desire, reward, and motivational salience. Dopamine facilitates sexual interest through mesolimbic and hypothalamic pathways, whereas D2 blockade may reduce libido and indirectly worsen arousal and orgasmic function (4, 6, 27). This is particularly important for antipsychotics with strong D2 antagonism, but it may also occur indirectly during antidepressant treatment when excessive serotonergic activity suppresses dopaminergic signaling. The relatively favorable sexual profiles of bupropion and aripiprazole are biologically coherent with this model: bupropion enhances noradrenergic-dopaminergic transmission, while aripiprazole, through partial D2 agonism, is less likely than pure D2 antagonists to cause profound dopaminergic suppression and may improve antipsychotic-related sexual adverse effects in some patients (1, 2, 4, 6, 27).

Endocrine mechanisms, particularly hyperprolactinemia, are most strongly implicated in antipsychotic-associated sexual dysfunction. D2 blockade in the tuberoinfundibular pathway increases prolactin release, which may suppress gonadal hormone production and contribute to decreased libido, erectile dysfunction, impaired lubrication, anorgasmia, menstrual irregularities, galactorrhea, gynecomastia, and fertility-related distress (3, 4, 6, 27, 28). However, prolactin is not a complete explanation for all cases. Sexual dysfunction may occur with only modest prolactin elevation, while some patients with marked hyperprolactinemia report limited sexual symptoms, suggesting that endocrine effects interact with receptor-mediated, illness-related, and psychosocial mechanisms (3, 4, 6, 27, 28).

Autonomic and vascular pathways further shape the clinical phenotype. Sexual arousal depends on intact parasympathetic and vascular responses, including nitric oxide-mediated vasodilation and genital blood flow. Anticholinergic effects may impair lubrication and genital arousal, while alpha-1 adrenergic antagonism may contribute to erectile difficulty, ejaculatory disturbance, or, more rarely, priapism (4, 6, 27). Antipsychotic-related endothelial and nitric oxide effects may also contribute to erectile dysfunction independently of prolactin, which helps explain why some patients report erectile impairment even when libido is relatively preserved (4, 6, 27).

Finally, these mechanisms are modified by nonspecific treatment effects such as sedation, emotional blunting, weight gain, metabolic deterioration, and reduced vitality, all of which may secondarily impair sexual functioning even when direct receptor-mediated toxicity is modest (1, 4, 6, 27). In practice, psychotropic-related sexual dysfunction arises from the interaction between drug pharmacology, psychiatric illness, endocrine vulnerability, physical health, and interpersonal context. This supports a domain-based and individualized approach to assessment and management.

### Assessment in clinical practice: baseline evaluation, direct questioning, and validated instruments

Sexual functioning should be assessed before treatment initiation whenever feasible, reassessed after dose titration or within the first 4–8 weeks of treatment, and reviewed again after dose changes, relapse, remission, or the emergence of new sexual symptoms (1–6). Reliance on spontaneous reporting is inadequate because patients frequently avoid raising sexual concerns unless asked directly (1, 3–6). Baseline assessment is essential for distinguishing illness-related dysfunction from treatment-emergent worsening.

In clinical practice, several patterns should be considered. Symptoms present before treatment and improving in parallel with psychiatric recovery are more likely to be predominantly illness-related. Persistent symptoms despite symptomatic improvement may reflect residual illness, comorbidity, relational factors, or medication effects. New symptoms emerging after treatment initiation or dose escalation, particularly orgasmic delay with serotonergic antidepressants or prolactin-related symptoms during antipsychotic treatment, increase the likelihood of treatment-emergent dysfunction. In many patients, however, sexual dysfunction reflects a combination of baseline vulnerability and medication-related worsening.

A concise assessment should document the affected domains of sexual function, including desire, arousal, erection or lubrication, orgasm or ejaculation, satisfaction, and pain; their temporal relationship to medication exposure and dose changes; psychiatric symptom control; partner and relational factors; substance use; comorbid medical illness; endocrine factors; and concomitant medication. Sex-specific factors, including menopausal status, reproductive stage, hormonal contraception, vascular risk factors, and the distinction between erectile dysfunction and lubrication or arousal difficulties, should also be considered when clinically relevant.

Validated instruments may improve detection and longitudinal comparison. The Arizona Sexual Experience Scale (ASEX) is a brief five-item instrument and is practical for routine screening. The Changes in Sexual Functioning Questionnaire (CSFQ) provides a broader domain-based assessment, whereas the Psychotropic-Related Sexual Dysfunction Questionnaire (PRSexDQ-SALSEX) and Antipsychotics and Sexual Functioning Questionnaire (ASFQ) are particularly useful when psychotropic-related dysfunction is suspected. The International Index of Erectile Function (IIEF) is most informative when erectile dysfunction is the predominant concern, while female-specific instruments may be appropriate for more detailed assessment of desire, arousal, lubrication, orgasm, or pain (38–41). Instrument selection should reflect the clinical question, consultation time, and local availability or licensing requirements.

Direct, neutral questioning is usually sufficient to open the discussion. Asking whether sexual desire, arousal, erection or lubrication, orgasm, ejaculation, or sexual satisfaction have changed since treatment initiation helps normalize the issue, improves detection, and reduces the risk that sexual adverse effects are misinterpreted as non-adherence, relapse, or lack of motivation.

### Management strategies: prevention, dose adjustment, switching, and adjunctive approaches

Management should begin with confirmation of the temporal relationship between symptoms and treatment, assessment of clinical severity and distress, and identification of potentially reversible contributors. These include incomplete remission of depression or psychosis, anxiety, endocrine abnormalities, vascular or metabolic disease, concomitant medication, alcohol or recreational substance use, and relational difficulties (1–6). Persistent or distressing dysfunction warrants active management because it may compromise adherence and recovery.

Prevention is preferable whenever sexual functioning is a major patient priority. When clinically appropriate, antidepressants with comparatively lower sexual burden, such as bupropion, mirtazapine, vortioxetine, agomelatine, moclobemide, vilazodone, or possibly tianeptine, may be considered (1, 2, 5, 12, 19–26). For antipsychotic treatment, prolactin-sparing options should be considered when efficacy, indication, and prior response permit. Aripiprazole has the strongest direct evidence for a favorable sexual and prolactin profile, whereas evidence for brexpiprazole, cariprazine, and lurasidone remains more limited (3, 4, 6, 27–34).

Dose reduction may be considered when dysfunction is dose-related and psychiatric symptoms are in stable remission, but it should be undertaken cautiously because symptom recurrence may outweigh gains in sexual tolerability (1, 5, 6). Switching is generally preferable when sexual dysfunction is moderate to severe, threatens adherence, or occurs in a patient who has achieved only partial benefit from the implicated medication. For antidepressants, switching from an SSRI, venlafaxine, or clomipramine to a lower-burden agent may be reasonable. For antipsychotics, switching from a prolactin-elevating agent to aripiprazole or another prolactin-sparing antipsychotic is usually preferred when psychiatric stability allows (1, 5, 6, 19, 42–50).

Adjunctive treatment may be more appropriate when the current regimen has provided substantial psychiatric benefit and switching is judged to carry a meaningful risk of relapse. Bupropion augmentation has the most consistent support among antidepressant-related strategies, particularly for reduced desire, arousal, and orgasmic difficulties; randomized studies have commonly evaluated sustained-release bupropion at 150 mg twice daily, although dose selection should remain individualized (5, 37, 42). Phosphodiesterase-5 inhibitors may be considered when erectile dysfunction predominates and may also improve selected domains of antidepressant-associated dysfunction in women, particularly orgasmic impairment (5, 37, 42). They do not directly treat low desire or anorgasmia and require routine assessment of cardiovascular status, concomitant nitrate therapy, and other contraindications.

For antipsychotic-associated hyperprolactinemia and sexual dysfunction, adjunctive aripiprazole is a reasonable option when switching is not feasible or may jeopardize psychiatric stability (45, 47–50). Switching to aripiprazole monotherapy may offer greater overall benefit when a change in antipsychotic treatment is clinically acceptable, whereas adjunctive aripiprazole may be preferable for patients with a strong prior response to the current antipsychotic or a high perceived relapse risk. Prolactin results should be interpreted together with sexual symptoms, menstrual changes, galactorrhea, gynecomastia, fertility concerns, and psychiatric status rather than used as the sole determinant of management.

Drug holidays are generally not preferred because evidence is limited, benefit is inconsistent, and intermittent treatment may provoke discontinuation symptoms, symptom recurrence, or non-adherence; this concern is particularly relevant for short half-life serotonergic antidepressants and for antipsychotic treatment (4, 5, 42). Psychoeducation, supportive psychotherapy, couples-oriented interventions, and sex therapy may be useful when shame, avoidance, performance anxiety, relationship distress, or maladaptive beliefs contribute to the clinical burden. Their evidence base remains less robust than that for pharmacological strategies, but these approaches are particularly relevant when dysfunction is multifactorial or persists despite medication adjustment (3, 5, 6).

A pragmatic clinical approach to assessment and management is summarized in **Table 2**.

**Table 2:** Practical assessment and management of psychotropic-related sexual dysfunction.

Clinical situation	Key assessment point	Likely contributors	Preferred action	Important caution
Before treatment initiation	Record baseline sexual function, distress, medical and relational factors	Psychiatric illness, endocrine or vascular factors, substance use, concomitant medication	Document baseline; discuss sexual tolerability when selecting treatment	Do not attribute pre-existing dysfunction to subsequent treatment
New symptoms after antidepressant initiation or dose increase	Timing, affected domain, psychiatric response	Serotonergic effects, especially with SSRIs, venlafaxine, or clomipramine	Reassess after titration or within 4–8 weeks; consider dose reduction, switching, or augmentation if persistent	Consider residual depression and other confounders
New symptoms after antipsychotic initiation or dose increase	Temporal relationship, prolactin-related symptoms, menstrual or endocrine changes	D2 blockade, hyperprolactinemia, autonomic effects	Consider switching to a prolactin-sparing agent or adjunctive aripiprazole	Psychiatric stability remains the primary consideration
Predominant loss of desire	Depression severity, prolactin, motivation, relationship context	Depression, serotonergic inhibition, dopaminergic suppression, hyperprolactinemia	Address illness-related and relational factors; consider a lower-burden agent or bupropion augmentation	Desire may improve with psychiatric remission alone
Orgasmic delay or anorgasmia	Onset after treatment, severity, distress	SSRIs, venlafaxine, clomipramine	Consider dose reduction, switching, or selected adjunctive treatment	Drug holidays are generally not preferred
Erectile dysfunction	Desire, vascular risk, endocrine status, medication timing	Autonomic, vascular, endocrine, and medication-related factors	Consider switching; use PDE-5 inhibitors when clinically appropriate	PDE-5 inhibitors do not directly treat low desire or anorgasmia
Lubrication or arousal difficulties	Hormonal status, reproductive stage, menopausal status, genital symptoms	Serotonergic or anticholinergic effects, endocrine factors	Consider dose adjustment, switching, and symptomatic local measures	Avoid reducing the assessment to libido alone
Hyperprolactinemia with sexual symptoms	Prolactin level, galactorrhea, menstrual changes, gynecomastia, fertility concerns	Risperidone, paliperidone, amisulpride, first-generation antipsychotics	Switch when feasible or add aripiprazole	Clinical symptoms and psychiatric status matter more than prolactin level alone
Persistent dysfunction after antidepressant discontinuation	Pre-treatment baseline, symptom onset, persistence after remission, alternative causes	Residual illness, medical factors, possible PSSD	Re-evaluate systematically and manage symptomatically	Avoid premature causal attribution, but do not dismiss persistent symptoms

Sexual function should be reassessed after dose titration, when new symptoms emerge, and after changes in psychiatric status or medication exposure. Management should be based on symptom domain, psychiatric stability, patient priorities, and the strength of available evidence.

### Special considerations and future directions

Several areas require cautious interpretation. Post-SSRI sexual dysfunction (PSSD) has received increasing attention as a potential persistent adverse outcome following serotonergic antidepressant exposure. Proposed diagnostic criteria emphasize the persistence of genital sensory changes, reduced libido, orgasmic impairment, erectile dysfunction, or lubrication difficulties after treatment discontinuation, provided that alternative medical, psychiatric, and pharmacological explanations have been carefully considered (43, 44, 51–53). However, its incidence, mechanisms, and risk factors remain uncertain. Available prevalence estimates are limited by under-recognition, retrospective reporting, inconsistent definitions, and the difficulty of distinguishing persistent treatment-related symptoms from residual depression, anxiety, medical conditions, or concurrent medication effects (43, 44, 51–53). PSSD should therefore be considered when symptoms begin during or shortly after serotonergic antidepressant exposure and persist beyond discontinuation. It may be addressed as part of balanced informed consent, although current evidence is insufficient to use the potential risk of PSSD as a stand-alone determinant of initial drug selection.

The evidence base also remains uneven across psychotropic agents. Some older medications and several newer compounds are clinically relevant but supported mainly by smaller trials, *post-hoc* analyses, pharmacovigilance data, endocrine findings, or mechanistic inference rather than direct comparative sexual-function studies. Comparative statements should therefore be interpreted as a pragmatic clinical synthesis rather than a formal quantitative ranking. Future studies should incorporate baseline sexual-function assessment before treatment initiation, reassessment after dose titration and when new symptoms emerge, validated instruments, adequate follow-up, dose information, sex-specific analyses, and outcomes that include distress, adherence, intimate relationships, fertility concerns, and quality of life.

Pharmacogenetic research suggests that interindividual differences in serotonergic, dopaminergic, glutamatergic, and drug-metabolism pathways may contribute to vulnerability to sexual adverse effects (54–56). However, the available findings remain preliminary, are not sufficiently replicated across populations, and do not currently support routine genetic screening to guide antidepressant or antipsychotic selection. Their principal value at present is to support future individualized risk stratification, particularly for patients with recurrent or severe treatment-emergent sexual dysfunction.

## Discussion

Psychotropic-related sexual dysfunction remains a frequent and clinically meaningful problem because it can affect quality of life, intimate relationships, self-esteem, and adherence (1–6, 19, 20). Its interpretation is complex: sexual dysfunction may predate treatment, persist as a residual symptom of illness, or worsen after pharmacological exposure (1–4, 7–11).

The central implication of this review is that sexual dysfunction should be treated as a multidimensional outcome rather than a unitary adverse effect. Antidepressants and antipsychotics may affect desire, arousal, orgasm or ejaculation, genital response, and subjective distress through partially overlapping serotonergic, dopaminergic, endocrine, autonomic, vascular, sedative, and metabolic pathways (1–6). This mechanistic diversity explains why no single management strategy is universally effective.

A second implication is that recognition depends on systematic assessment. Brief baseline documentation, direct follow-up questioning, and selective use of validated instruments are usually sufficient to identify clinically relevant dysfunction and guide management (1, 2, 4, 38–41).

Management should remain individualized. Prevention through informed drug selection is preferable when sexual tolerability is likely to influence adherence, while dose adjustment, switching, or adjunctive treatment may be appropriate when dysfunction emerges (2, 6, 17, 42–50). The choice should depend on psychiatric stability, symptom domain, patient priorities, and the strength of evidence for the proposed intervention.

Several limitations should be acknowledged. Comparative estimates vary according to study design, illness stage, baseline dysfunction, assessment method, dose, comorbidity, and polypharmacy (1, 2, 4–6, 28). Short-term trials may underestimate longer-term burden, while real-world studies are vulnerable to confounding. Persistent post-treatment dysfunction and pharmacogenetic vulnerability also remain incompletely understood and require further longitudinal research (51–56).

## Conclusion

Sexual dysfunction should be treated as a core tolerability and adherence outcome in psychopharmacology. Before initiating antidepressant or antipsychotic treatment, clinicians should document baseline sexual functioning, discuss patient priorities, and consider drug-specific sexual tolerability when several clinically appropriate options exist.

Higher-risk patterns are most consistently associated with strongly serotonergic antidepressants and prolactin-elevating antipsychotics. Lower-burden options exist, but the evidence is uneven and must be balanced against psychiatric efficacy, previous response, comorbidity, and patient preference. Routine direct questioning, validated assessment tools, dose optimization, switching, targeted adjunctive treatment, and psychosocial interventions provide a practical framework for individualized care.
